# Strength deficits of the shoulder complex during isokinetic testing in people with chronic stroke

**DOI:** 10.1590/bjpt-rbf.2014.0030

**Published:** 2014

**Authors:** Lucas R. Nascimento, Luci F. Teixeira-Salmela, Janaine C. Polese, Louise Ada, Christina D. C. M. Faria, Glória E. C. Laurentino

**Affiliations:** 1 Discipline of Physiotherapy, Faculty of Health Sciences, The University of Sydney, Sydney (NSW), Australia; 2 Department of Physical Therapy, Universidade Federal de Minas Gerais (UFMG), Belo Horizonte, MG, Brazil; 3 Department of Physical Therapy, Universidade Federal de Pernambuco (UFPE), Recife, PE, Brazil

**Keywords:** cerebrovascular disease, hemiparesis, shoulder complex, muscle strength, physical therapy

## Abstract

**OBJECTIVES:**

To examine the strength deficits of the shoulder complex after stroke and to characterize the pattern of weakness according to type of movement and type of isokinetic parameter.

**METHOD:**

Twelve chronic stroke survivors and 12 age-matched healthy controls had their shoulder strength measured using a Biodex isokinetic dynamometer. Concentric measures of peak torque and work during shoulder movements were obtained in random order at speeds of 60°/s for both groups and sides. Type of movement was defined as scapulothoracic (protraction and retraction), glenohumeral (shoulder internal and external rotation) or combined (shoulder flexion and extension). Type of isokinetic parameter was defined as maximum (peak torque) or sustained (work). Strength deficits were calculated using the control group as reference.

**RESULTS:**

The average strength deficit for the paretic upper limb was 52% for peak torque and 56% for work. Decreases observed in the non-paretic shoulder were 21% and 22%, respectively. Strength deficit of the scapulothoracic muscles was similar to the glenohumeral muscles, with a mean difference of 6% (95% CI -5 to 17). Ability to sustain torque throughout a given range of motion was decreased as much as the peak torque, with a mean difference of 4% (95% CI -2 to 10).

**CONCLUSIONS:**

The findings suggest that people after stroke might benefit from strengthening exercises directed at the paretic scapulothoracic muscles in addition to exercises of arm elevation. Clinicians should also prescribe different exercises to improve the ability to generate force and the ability to sustain the torque during a specific range of motion.

## Introduction

Stroke is one of the leading causes of disability worldwide and has a significant impact on physical, emotional, and social lives^1^ ^,^ ^2^. It has been suggested that rehabilitation strategies designed to improve activity after stroke should be based upon understanding of the nature of the main impairments, as well as knowledge of their relative contributions to disabilities^3^. Studies aimed at increasing our understanding of the nature of upper limb impairments are necessary to underpin rehabilitation, considering that the upper limb is required for most activities of daily living^1^. In particular, shoulder movements are necessary to carry out activities like feeding, combing hair, and reaching overhead, thus a compromised shoulder complex could lead to limitations in several activities^3^ ^,^ ^4^. Previous studies indicated that muscular weakness is the most common impairment following stroke and has been shown to be significantly related to limitations during these upper limb activities^1^ ^,^ ^5^ ^,^ ^6^.

The shoulder complex exhibits the greatest amount of movement in the human body. This mobility is the result of the combined and constrained motions of two main joints, the glenohumeral and scapulothoracic^7^ ^,^ ^8^. Weakness in the scapulothoracic or the glenohumeral muscles may cause imbalances in the force couples around the shoulder complex, leading to abnormal kinematics^9^ ^,^ ^10^. Since these muscles are constrained to act as a single unit, any abnormality in one muscle may result in instability which, in turn, may decrease movement during upper limb activities^11^ ^,^ ^12^.

Previous studies^13^ ^,^ ^14^ on shoulder muscle weakness have measured isometric strength, which does not reflect the dynamic nature of the upper limb movements and is limited to one aspect of muscle strength (i.e. peak torque). Although peak torque is an excellent indicator of maximum strength, it does not take into account the ability to sustain a produced torque throughout a given range of motion (i.e. work)^15^. The ability to generate large muscle forces is of little functional benefit if the force cannot be sustained during the time required to perform an activity. Incomplete range of motion during activities of daily living is clinically evident in people after stroke and may be related to inability to sustain a produced torque.

Although previous studies have provided evidence that shoulder muscles are generally weak after stroke^13^ ^,^ ^14^ and that both peak torque and work are decreased during the abduction of the upper limb^3^, there is still no specific information regarding glenohumeral muscle weakness compared with scapulothoracic muscle weakness. Despite the fact that neurological rehabilitation has changed considerably over the past decades, strength training of the shoulder muscles is still uncommon, particularly strengthening of the scapulothoracic muscles. This information could help clinical practice since it has been suggested that strong scapulothoracic muscles are necessary to achieve adequate range of motion during arm elevation^11^.

Therefore, to understand the nature of the strength deficit of the shoulder muscles in people with stroke, this study aimed to investigate dynamic strength deficits according to type of movement and type of isokinetic parameter. Type of movement was defined as: scapulothoracic (protraction and retraction which predominantly involves movement of the scapula on the chest wall), glenohumeral (internal and external shoulder rotation which predominantly involves movement at the glenohumeral joint) or combined (shoulder flexion and extension which involves movement of the scapula and the glenohumeral joint). Type of isokinetic parameter was analyzed as: maximum strength (peak torque) or work (ability to produce and sustain torque throughout a given range of motion). The specific research questions were:

- Is the strength deficit during scapulothoracic movement less affected than during glenohumeral movement in people with stroke?
- Is the ability to sustain torque throughout a given range of motion less affected than maximum strength?

The findings will provide information regarding the nature of weakness following stroke. Examining different parameters of strength of stable chronic individuals after stroke will help guide clinical practice by suggesting specific muscles and strength parameters to be targered with strengthening interventions during rehabilitation of both acute and chronic patients.

## Method

### Participants

Twelve chronic stroke survivors and 12 healthy controls were recruited from the general community of the city of Belo Horizonte, Brazil. Participants with stroke were included if they: were >20 years old; had a time since the onset of unilateral stroke greater than six months; had no pain or contractures of the upper limb joints which could prevent the test procedures; had no cognitive deficits (scores>24 out of 30 on the Mini-mental state examination)^16^; had mild or moderate upper limb motor impairments (scores between 30-65 out of 66 on the Fugl-Meyer - upper limb scale)^17^; had mild or moderate increases in muscle tone of the elbow flexors (scores <3 out of 4 on the Modified Ashworth Scale)^18^; and had no other neurological or orthopedic disorders. Healthy participants matched by age, gender, and upper limb dominance were included if they had no cognitive deficits. This study was approved by the University's Ethical Review Board, *Universidade Federal de Minas Gerais* (UFMG), Belo Horizonte, MG, Brazil (ETIC 0539.0.203.000-09), and all the participants signed the consent forms.

### Procedures

The participants attended the university laboratory on one occasion, for about 90 minutes. First, both groups provided consent prior to data collection and background information regarding their age, gender, body mass, height, cognition, and grip strength. Grip strength was measured using a Jamar dynamometer^19^, and the average value after three repetitions was recorded. The time since the onset of stroke, the paretic side, motor impairments, muscle tone, and amount and quality of use of the paretic upper limb using the Motor Activity Log^20^ were also collected for the stroke group for descriptive purposes.

Then, peak torque and work were obtained during the movements of scapular protraction and retraction; external and internal shoulder rotation; and shoulder flexion and extension. The order of testing of the movements was randomized. After a brief explanation, participants executed three sub-maximal familiarization trials, followed by five maximal concentric-concentric trials for each evaluated movement. The non-paretic side of the stroke and the dominant side of the control participants were tested first. During the tests, blood pressure and heart rate were constantly monitored, and standardized procedures were employed by having the same physical therapist collecting all of the data.

### Measurement of strength of the shoulder complex

Strength of the shoulder complex was measured as peak torque and work obtained with the Biodex isokinetic dynamometer (Biodex Medical System 3 Pro, Shirley, NY, USA) at a speed of 60°/s. The dynamometer was calibrated following the manufacturer's recommendations and the axis of the dynamometer was aligned with that of each specific joint^21^ ^,^ ^22^, and the six movements were evaluated^9^ ^,^ ^23^ ^,^ ^24^. Modifications of the testing positions and ranges of motion were performed to minimize possible compensatory movements^25^. Gravity corrections were employed during the tested movements, except for the scapular protraction and retraction movements, since these movements are performed in the horizontal plane^9^.

For the scapular protraction and retraction movements, the closed chain attachment was fixed to the dynamometer in the horizontal position. The dynamometer shaft was rotated 30º, and the participants were seated with their arms in the scapular plane^9^ ^,^ ^26^. The elbow was kept extended by a stabilizing device and the trunk was stabilized by two crossed straps. Movement was performed at 12.2 cm/s from 20º of protraction to 10º of scapular retraction.

For the external and internal shoulder rotation movements, the participants were positioned in supine position to reduce the scapulothoracic movements, with 90º of shoulder abduction and elbow flexion. The rotation axis of the dynamometer was aligned to the shoulder joint according to Moraes et al.^22^, and movement was performed within an arc of 90º, between 40º of external rotation and 50º of internal rotation^22^. This range of motion was chosen to prevent passive restriction of the rotator cuff and the possible concurrent onset of pain^24^.

For the shoulder flexion and extension movements, the participants were seated with the elbow in extension and movement was performed within an arc of 90°, between 20º of shoulder extension and 70º of flexion. The rotation axis of the dynamometer was aligned to the shoulder joint according to Kim et al.^23^.

### Data reduction

Strength was measured both as peak torque and work. Peak torque is the product of mass, acceleration, and the lever arm length^15^. Although peak torque is an excellent indicator of maximum strength, it does not take into account the range of motion. For this reason, work was also calculated to indicate the ability to produce and sustain torque throughout a given range of motion^15^. Peak torque was the maximum torque produced during five trials, and the total work was the cumulative amount of work produced by the participants during several trials. Both peak torque (Nm/s) and work (J) were normalized by body mass.

Strength deficits were calculated using the control group as a reference, according to Alon^27^, as follows: Deficit = 100 - (stroke/control * 100). Therefore, the pattern of strength could be examined across three different experimental conditions regarding the type of movement: predominantly scapulothoracic (protraction and retraction), predominantly glenohumeral (internal and external shoulder rotations), and combined glenohumeral and scapulothoracic movements (shoulder flexion and extension); and between two experimental conditions regarding the type of isokinetic parameter: maximum strength and work.

### Data analysis

Descriptive statistics, tests for normality (Shapiro-Wilk), and homogeneity of variance (Levene) were carried out for all outcome variables, using the SPSS for Windows software (SPSS, Chicago, IL). Multifactorial repeated measures ANOVA were employed to investigate differences in the strength deficits across the three experimental conditions related to type of movement (predominantly scapulothoracic, predominantly glenohumeral, and combined glenohumeral and scapulothoracic movements). Paired t-tests were employed to compare differences between the two types of isokinetic parameters (peak torque and work). Significance level was set at α=0.05. Mean differences were calculated and were provided with their 95% confidence intervals (95% CI).

## Results

### Participants

As shown in Table 1, the stroke group was comprised of 12 individuals (six men) with a mean age of 52 years (SD 11, range 32 to 67 years), and a mean time since the onset of stroke of 10 years (SD 4.9). The control group was comprised of 12 volunteers with a mean age of 52 years (SD 12, range 30 to 66 years), matched by age, gender and hand dominance.

**Table 1.** Characteristics of the participants.

Characteristic	Stroke *n*=12	Control *n*=12
Age *(years)*, mean (SD)	52.0 (10.5)	51.8 (11.8)
Gender, n male (%)	6 (50)	6 (50)
Body mass *(kg)*, mean (SD)	73.7 (10.4)	69.8 (13.7)
Height *(m),* mean (SD)	1.65 (0.1)	1.68 (0.1)
Cognition *(MMSE 0-30)*, mean (SD)	27.5 (2.0)	28.8 (1.7)
Grip strength – paretic* (Nm)*, mean (SD)	14.9 (10.4)	35.5 (9.5)
Grip strength – non-paretic* (Nm)*, mean (SD)	33.0 (9.3)	37.6 (9.7)
Time since stroke *(years)*, mean (SD)	10.0 (4.9)	NA
Side of hemiparesis, n right (%)	7 (58)	NA
Motor impairments *(Fugl-Meyer UL 0-66)*, mean (SD)	47 (10)	NA
Muscle tone *(*Modified Ashworth scale 0-4), n (%)		
0	3 (25)	NA
1	3 (25)	NA
1+	1 (8)	NA
2	2 (17)	NA
3	3 (25)	NA
Amount of UL use *(MAL 0-5)*, mean (SD)	3.4 (1.5)	NA
Quality of UL use *(MAL 0-5)*, mean (SD)	3.4 (1.6)	NA

MMSE =Mini-mental state examination

UL =upper limb

MAL =Motor Activity Log

NA =not applicable

Table 2 provides the magnitude of strength for both groups and sides, and the strength deficit for each evaluated movement. The average deficit in peak torque was 52% (ranging from 41 to 57%) for the paretic upper limb and 21% (ranging from 13 to 34%) for the non-paretic upper limb. The average deficit in work measures was 56% (ranging from 48 to 62%) for the paretic upper limb and 22% (ranging from 13 to 29%) for the non-paretic upper limb.

**Table 2.** Mean (SD) peak torque (Nm/s) and work (J) for each side of each group and mean (SD) strength deficit for each side of stroke group as a % of control group.

	Strength						Strength deficit*	
	Stroke			Control			Stroke	
	Paretic	Non-paretic		Dominant	Non-dominant		Paretic	Non-paretic
Peak torque								
Shoulder internal rotation	18 (7)	31 (7)		42 (12)	42 (11)		52 (20)	22 (19)
Shoulder external rotation	18 (7)	31 (5)		36 (7)	37 (8)		46 (25)	13 (18)
Shoulder flexion	43 (14)	64 (27)		8 (29)	84 (36)		41 (26)	18 (30)
Shoulder extension	30 (11)	57 (18)		69 (15)	72 (15)		55 (18)	18 (22)
Scapular protraction	172 (71)	312 (95)		435 (114)	444 (112)		57 (20)	25 (29)
Scapular retraction	214 (75)	335 (99)		509 (116)	522 (126)		55 (20)	34 (13)
Work								
Shoulder internal rotation	21 (10)	38 (11)		52 (17)	53 (17)		57 (23)	26 (18)
Shoulder external rotation	21 (10)	39 (10)		46 (10)	46 (10)		50 (30)	13 (20)
Shoulder flexion	41 (14)	69 (25)		94 (37)	92 (36)		48 (28)	22 (27)
Shoulder extension	29 (14)	61 (21)		80 (20)	80 (22)		62 (20)	22 (24)
Scapular protraction	63 (21)	144 (32)		187 (55)	182 (50)		61 (18)	22 (8)
Scapular retraction	82 (29)	152 (33)		219 (49)	215 (45)		59 (15)	29 (12)

* Strength deficit = 100 - (stroke/control x 100).

### Pattern of strength deficit according to type of movement

Table 3 provides the strength deficit of the paretic upper limb and the mean difference between the types of movement. There were no significant differences in strength deficits between the three different types of movement regarding peak torque (*F*=2.96, *p*=0.08) and work (*F*=1.45, *p*=0.26). The average mean difference between scapulothoracic deficit and the glenohumeral deficit was 6% (95%CI -5 to 17), and the average mean difference between scapulothoracic deficit and the combined deficit was 6% (95%CI -6 to 18).

**Table 3.** Mean (SD) strength deficit* of stroke group as a % of control group for each type of movement and mean differences (95%CI) between types of movement.

Isokinetic parameter	Type of movement			Difference between types of movement		
	Scapulothoracic	Glenohumeral	Combined	Scapulothoracic minus glenohumeral	Scapulothoracic minus combined	Glenohumeral minus combined
Peak torque	56 (20)	50 (18)	50 (19)	6 (-4 to 16)	6 (-1 to 13)	0 (-9 to 9)
Work	60 (16)	53 (26)	55 (22)	6 (-7 to 20)	5 (-6 to 16)	-1 (-11 to 9)
**Average**	**58 (18)**	**51 (22)**	**52 (20)**	**6 (-5 to 17)**	**6 (-6 to 18)**	**0 (-10 to 9)**

* Strength deficit = 100 - (stroke/control x 100).

### Pattern of strength deficit according to type of isokinetic parameter

Table 4 provides the strength deficit of the paretic upper limb and the mean difference between the types of isokinetic parameter. There were no significant differences in strength deficits between the two different of types isokinetic parameter during the scapulothoracic movements (t=1.35, p=0.20) and the glenohumeral movements (t=0.83, p=0.42). A significant difference in strength deficit between the two different types of isokinetic parameter was found during the combined movement (t=2.8, p=0.02), with a mean difference of 5% (95% CI 1 to 9). Overall, there was no significant difference between types of isokinetic parameter, with an average mean difference between peak torque deficit and work deficit of 4% (95% CI -2 to 10) for the paretic upper limb.

**Table 4.** Mean (SD) strength deficit of stroke group* as a % of control group and mean differences (95%CI) between types of isokinetic parameters.

Type of movement	Type of isokinetic parameter		Difference between types of isokinetic parameter
	Work	Peak torque	Work minus peak torque
Scapulothoracic	60 (16)	56 (20)	4 (-3 to 11)
Glenohumeral	53 (26)	50 (18)	3 (-6 to 12)
Combined	55 (22)	50 (19)	5 (1 to 9)
**Average**	**56 (21)**	**52 (19)**	**4 (-2 to 10)**

* Strength deficit = 100 - (stroke/control x 100).

## Discussion

This is the first study to measure dynamic strength of the shoulder complex in people with stroke during different movements. Strength deficits in peak torque and work during six movements of the shoulder complex were calculated, so that the pattern of weakness could be examined according to type of movement and type of isokinetic parameter. In terms of the type of movement, the results indicate that the strength deficit in the scapulothoracic muscles is the same as the strength deficit in the glenohumeral muscles in people with chronic stroke. In addition, in terms of the type of isokinetic parameter, the results indicate that the deficit in the ability to sustain a contraction throughout a given range of motion is the same as the deficit in the ability to produce maximal force.

During arm elevation, glenohumeral and scapulothoracic motion occurs synchronously in about a 2:1 overall ratio, with glenohumeral motion occurring alone during the first 30º of elevation^8^ ^,^ ^11^. Strength deficits in scapulothoracic movement (protraction and retraction) were similar to deficits in glenohumeral movement (internal and external shoulder rotations). This suggested that deficits in strength of scapulothoracic and glenohumeral muscles might be equally important in terms of explaning the inability to elevate the upper limb following stroke.

The ability to sustain a contraction was as decreased as the ability to produce maximal force during both scapulothoracic and glenohumeral movements, which suggests that even if upper limb movements are initiated, the inability to sustain torque may compromise the execution of movements after stroke. Thus, people after stroke may get into a vicious cycle, in which weakness limits arm elevation and subsequent inactivity increases this weakness. Although a significant difference between types of isokinetic parameter was found during the combined movement, the mean difference was only 5% which does not appear to be clinically important. Considering that both types of isokinetic parameter are largely decreased in comparison with the control group, it is recommended that strengthening interventions directed at the shoulder complex focus on both parameters: maximum strength and work.

While weakness of the shoulder muscles has been previously reported using isometric measurements^13^ ^,^ ^14^, examination of dynamic strength of scapulothoracic movements has not been investigated. The scapula plays a critical role in controlling the position of the glenoid fossa and maintaining optimal length-tension relationships during upper limb elevation^26^. Therefore, relatively small changes in strength of the scapulothoracic muscles may affect its alignment and compromise upper limb movements^7^ ^,^ ^28^ ^,^ ^29^. Cools et al.^9^ reported significant weakness of protraction strength in athletes with impingement symptoms and difficullty with overhead movement. This supports the hypothesis that scapulothoracic muscle weakness may be related to shoulder disabilities.

The paretic side was weaker than the non-paretic side in the stroke group regardless the type of movement and type of isokinetic parameter. These results are in accordance with previous studies that measured muscle strength in both paretic and non-paretic sides after stroke^14^ ^,^ ^30^ ^,^ ^31^. In the present study, strength deficits of the non-paretic side were less than half than those of the paretic side. Although a decrease in force production has been described in the non-paretic side, deficits were obviously not large enough to be clinically relevant, since even severely disabled stroke subjects do not complain about weakness on their non-paretic side. The results of this study are in accordance with Avila et al.^3^, who described a significant decrease in peak torque and work in the paretic upper limb during shoulder abduction and a non-significant decrease in the non-paretic upper limb of individuals with stroke compared with a control group.

A limitation of this study was the narrow range of motion used to measure protraction and retraction movements. However, this was done to minimize possible compensatory trunk movements and recruitment of stronger muscles. Althought the mean time frame post-stroke varied, it reflects the characteristics of the stroke population found in the community, and potential confounding factors were minimized by matching with healthy subjects. However, future studies with a wider range of severity of impairments are necessary to enhance the generalizability of these findings for the whole stroke population. Since the present results reflected the concentric muscular performance of people with mild-to-moderate upper limb impairments, caution should be taken to extrapolate the results to individuals with severe chronic stroke.

There are important clinical implications related to the findings that the strength deficits of the scapulothoracic muscles were the same as the deficits of the glenohumeral muscles and that the inability to sustain a contraction throughout a given range of motion was the same as the inability to produce a maximal force. These findings suggest that people with stroke might benefit from strengthening exercises specifically directed at the scapulothoracic muscles (i.e. protraction and retraction) and the glenohumeral muscles (i.e. external and internal rotation) in the early stages, so that both muscle groups are strengthened. Then, arm elevation exercises that combine both sets of muscle groups can be initiated and may be more successful since arm elevation relies on a combination of scapulothoracic and glenohumeral movements. Furthermore, strengthening exercises should include both fast and sustained contractions.

Since the muscles around the shoulder complex act in synergy, restitution of the appropriate balance between scapulothoracic and glenohumeral muscles might increase their synergic actions, thereby improving the ability to perform activities of daily living^7^ ^,^ ^22^. Therefore, activities that require arm elevation could be combined with strength training to allow the targered muscles in the rehabilitation program to improve the scapulohumeral rhythm and guarantee appropriate range of motion in daily activities^32^ ^,^ ^33^.

## Conclusions

The present results indicate that people with stroke who have mild to moderate upper limb impairments demonstrate clinically significant weakness of the paretic shoulder and suggest a non-significant weakness of the non-paretic upper limb compared to healthy controls. There were no distinct patterns of strength deficits in terms of type of movement, with equal deficits in movements which were predominantly scapulothoracic and glenohumeral. These findings suggest that people with stroke might benefit from strengthening exercises directed at both the scapulothoracic and the glenohumeral muscles. Similarly, there were no distinct patterns of strength deficits in terms of type of isokinetic parameters, with equal deficits regarding maximal strength and the ability to sustain a contraction throughout a given range of motion. These findings suggest that clinicians should prescribe strengthening exercises to increase the ability to generate force and to sustain the torque during a specific movement or range of motion. Randomized trials are necessary to verify the efficacy of strengthening both at the scapular and glenohumeral muscles during early rehabilitation in improving upper limb activities.
